# Uncontrolled Hypertension Leading to the Diagnosis of Renal Cell Carcinoma: A Case Report

**DOI:** 10.7759/cureus.109863

**Published:** 2026-05-29

**Authors:** João Loja, Rafael Ferreira Nascimento, Elisa Caldeira, Maria Eduarda Moniz, João Miguel Freitas

**Affiliations:** 1 Internal Medicine, Serviço de Saúde da Região Autónoma da Madeira, Entidade Pública Empresarial da Região Autónoma da Madeira (SESARAM, EPERAM), Funchal, PRT; 2 Internal Medicine, Hospital Central do Funchal, Funchal, PRT

**Keywords:** acute otitis media, holistic approach, hypertension, nephrectomy, renal cell carcinoma

## Abstract

Renal cell carcinoma (RCC) is frequently diagnosed unexpectedly, as early disease usually lacks clinical findings. Although hypertension is a risk factor for RCC, it may also represent a paraneoplastic manifestation of the disease.

We report the case of a man who presented to the emergency department with acute ear pain and was found to have severe, newly diagnosed hypertension. Despite multidrug antihypertensive therapy, blood pressure remained poorly controlled, prompting further investigation for secondary causes. Imaging studies revealed a cystic renal mass, and the patient underwent partial nephrectomy, with histopathological confirmation of RCC. After tumor resection, the patient maintained improved blood pressure control over time, allowing a reduction of his antihypertensive therapy.

This case highlights resistant hypertension as a potential manifestation of an underlying malignancy and underscores the importance of a comprehensive clinical approach in patients with poorly controlled hypertension.

## Introduction

RCC, also known as hypernephroma or Grawitz tumor, is the most common primary malignancy of the kidney [1]. The classic triad of flank pain, hematuria, and a palpable mass in the flank is observed in only a minority of patients and typically reflects advanced disease [2]. In contrast, more than half of RCC cases are currently diagnosed incidentally through imaging modalities such as ultrasound or computed tomography, often at an earlier stage [1].

Hypertension is reported in up to 40% of patients with RCC [3]. However, true paraneoplastic hypertension is considerably less common, with estimates ranging from approximately 2% to 15% [4]. Hypertension has a complex and bidirectional relationship with RCC [1]. Essential hypertension is a well-established risk factor for the development of this tumor [1]. Although the underlying mechanisms are not fully elucidated, proposed pathways include increased oxidative stress due to reactive oxygen species and overexpression of factors induced by chronic renal hypoxia [5]. Conversely, RCC may also cause secondary hypertension as a paraneoplastic manifestation [2]. Tumor-related renin secretion can lead to reversible hypertension [3]. Additional mechanisms include compression of the renal artery or its branches and the presence of arteriovenous fistulas [3].

Given that the classic clinical presentation of RCC often occurs at later stages, early recognition of paraneoplastic syndromes may play a crucial role in making a timely diagnosis [6].

We describe the case of a patient who was diagnosed with a kidney neoplasm after an exhaustive investigation of his difficult-to-manage hypertension.

## Case presentation

A 58-year-old Caucasian man went to the emergency service with a two-day history of acute left ear pain. He had a medical history notable for type 2 diabetes, obesity, and was a heavy smoker. His chronic medication regimen included metformin 850 mg twice daily and canagliflozin 100 mg once daily.

On admission, the physical examination revealed systolic and diastolic blood pressure values averaging 220/110 mmHg in both upper limbs, measured in standing and sitting positions. He reported no history of weight loss, sweating, headaches, visual disturbances, or neurological symptoms. Cardiovascular examination revealed a regular heart rate with no murmurs, rubs, or gallops. Peripheral pulses were symmetric, regular, and synchronous. The carotid pulse was regular, with a rate of 82 beats per minute. Abdominal examination was unremarkable, with no palpable masses, tenderness, or bruits. There were no clinical features suggestive of hypercortisolism. The kidneys were not palpable. Fundoscopic examinations disclosed no papilledema, and neither retinal hemorrhages nor microaneurysms were observed. Otoscopy revealed narrowing of the left external auditory canal, accompanied by hyperemia of the ipsilateral tympanic membrane. No other abnormalities were noted in the remainder of the physical examination. The patient persisted with markedly elevated blood pressure values despite oral antihypertensive therapy, prompting the initiation of an intravenous labetalol infusion. The initial laboratory workup and 12-lead electrocardiogram revealed no significant abnormalities. Cranial computed tomography (CT) showed no acute lesions. After stabilization of his blood pressure profile, he was given amoxicillin-clavulanic acid for acute otitis media and started azilsartan plus chlortalidone. He was referred to an internal medicine consultation for further evaluation of his hypertension. Initial laboratory investigation included a complete blood count, lipid profile, hemoglobin A1c, thyroid-stimulating hormone (TSH), and thyroxine (free T4), all of which were within normal ranges. Serum electrolytes, including potassium, were within normal limits. Serum creatinine and urea were normal. Hemogram and biochemistry are illustrated in Table 1. Urinalysis showed no hematuria or proteinuria (Table 2). 24-hour urinary cortisol was at normal levels. Urinary metanephrines were obtained and found to be within normal values. The aldosterone levels were normal, and plasma renin was slightly elevated, even though this measurement did not allow us to draw conclusions, since the patient was medicated with an angiotensin 2 receptor blocker. A tenuous elevation in plasma normetanephrine was observed but was not considered clinically significant, given the potential for false-positive results in the context of recent caffeine intake or tobacco use prior to testing. The hormonal study is summarized in Table 3.

**Table 1:** Initial hemogram and biochemistry (including lipid profile)

Markers	Results	Normal values
Leucocytes	10.9 x 10^3/µL	4.2 - 10.8
Neutrophils	6.8 x 10^3/µl	1.9 - 7.2
Platelets	215 x 10^3/µL	144.0 - 440.0
Hemoglobin	14.3 g/dL	13.7 - 17.3
Hemoglobin A1c	5.5 %	4.0 - 6.0
Urea	46 mg/dL	16.6 - 48.5
Creatinine	1.07 mg/dL	0.7 - 1.2
Sodium	138 mEq/L	136 - 145
Potassium	3.7	3.5 - 5.0
Chlorine	95 mg/dL	98.0 - 107.0
Total cholesterol	134 mg/dL	< 200
Cholesterol HDL	47 mg/dL	> 35
Cholesterol LDL	64 mg/dL	< 100

**Table 2:** 24-hour urine

Markers	Results	Normal results
Creatinine (urine 24h)	1807 mg/24h	1040 - 2350
Total proteins (urine 24H)	182 mg/24h	< 140

**Table 3:** Hormonal study of secondary hypertension

Markers	Results	Normal values
Aldosterone	18.8 ng/dL	1.0 - 31.0
Urinary cortisol	24 µg/24h	4.2 - 60.0
Vanillylmandelic acid (urine)	6.5 mg/24h	< 6.7
Homovalinic acid (urine)	4.5 mg/24h	< 6.2
5-hydroyindoleacetic acid (5-HIAA) (urine)	5.2 mg/24h	< 8.2
Plasma normetanephrine	211.1 pg/mL	< 180
Plasma metanephrine	38.5 pg/mL	< 90
Total plasma metanephrines	249.6 pg/mL	< 290
Urinary normetanephrine	385.7 µg/24h	< 778.6
Urinary metanephrine	152.1 µg/24h	< 374.7
Urinary 3-methoxytyramine	78.4 µg/24h	< 426.4
Total urinary metanephrines	616 µg/24h	< 1300
Renin	142.7 mU/L	4.2 - 45.0
Free thryroxine (Free T4)	1.4 ng/dL	0.6 - 1.7
Thyroid - Stimulating Hormone (TSH)	0.7 mlU/L	0.3 - 4.7

The transthoracic echocardiogram demonstrated preserved global systolic function, with no evidence of left ventricular hypertrophy or other structural abnormalities. The 24-hour ambulatory blood pressure monitoring confirmed values consistent with uncontrolled hypertension, despite the patient being treated with three antihypertensive drugs.

**Figure 1:**
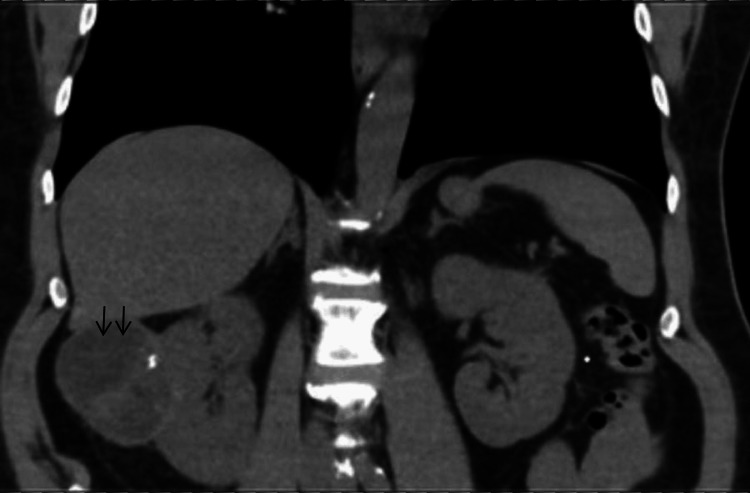
Abdominopelvic CT demonstrates a large multiloculated cystic lesion in the right kidney, with internal septations and calcifications, consistent with a complex renal cyst (arrows)

Given the persistence of elevated blood pressure values, amlodipine, bisoprolol, and methyldopa were added to his regimen, resulting in treatment with a total of five antihypertensive drugs. To further assess potential causes of renovascular hypertension, an abdominopelvic CT scan was performed, pointing to a multiseptated, loculated cyst with calcifications in the right kidney, measuring 8 cm in its greatest dimension (Figure 1).

The patient was subsequently followed in a urology consultation and later underwent a right partial nephrectomy. Histopathological examination confirmed renal cell carcinoma, clear cell subtype, with a cystic pattern, WHO/ISUP grade 1. The pathology report showed no invasion of the perirenal adipose tissue, no evidence of lymphovascular invasion, and negative surgical margins. After surgery, his antihypertensive regimen was adjusted, allowing a reduction to three medications. Blood pressure remained well controlled throughout the 24-month follow-up. The patient continued regular follow-up in both internal medicine and urology.

## Discussion

The patient had a history of obesity and was a chronic smoker, which are two risk factors for RCC [7]. Obesity has been associated with chronic renal hypoxia, which may promote tumorigenesis through activation of the vascular endothelial growth factor (VEGF) signaling pathway [8]. Several studies have shown that smokers have an increased risk of developing renal cell carcinoma, with an association that is dose-dependent, seen in both genders [8]. The complaint that motivated the patient to come to the emergency was acute ear pain. RCC can rarely metastasise to the temporal bone, leading to otologic symptoms such as otalgia, tinnitus, or hearing loss [9]. However, no evidence of metastatic disease was identified in this case, and otoscopic findings were consistent with acute otitis media, supporting an infectious rather than neoplastic etiology.

A notable feature of this case was the presence of resistant hypertension, which significantly improved following partial nephrectomy. This temporal relationship suggests a likely paraneoplastic mechanism [10]. RCC is known to be associated with a variety of paraneoplastic syndromes, observed in over half of patients, including hypertension, hypercalcemia, anemia, and fever [11]. These manifestations are thought to result from tumor-related secretion of hormones and cytokines [12]. Importantly, the resolution of paraneoplastic features after tumor removal may serve as an indicator of disease control, whereas persistence can suggest residual or recurrent disease [12]. In this patient, sustained blood pressure control following surgery further supports a possible paraneoplastic association between renal tumor and resistant hypertension [12].

Management of RCC is primarily guided by tumor staging [1,7]. In this case, the tumor was classified as stage I, for which partial nephrectomy is the preferred treatment and is often curative [7]. Alternative approaches for localized RCC include active surveillance and ablative techniques, depending on patient comorbidities and preferences [11]. Partial nephrectomy has the advantage of preserving renal function when compared to radical nephrectomy while offering comparable oncological outcomes in early-stage disease [13]. Therefore, it represents the standard of care for appropriately selected patients with localized RCC [13]. Systemic therapies such as immunotherapy or targeted therapies are available for advanced disease [1,11].

## Conclusions

This case emphasizes the importance of a holistic clinical approach, rather than focusing solely on the presenting complaint. It illustrates resistant hypertension as a potential paraneoplastic manifestation of renal cell carcinoma (RCC), with resolution following tumor resection.

Acknowledgement of secondary causes of hypertension, particularly in cases of weak therapeutic response, is essential and may significantly impact patient outcomes. In this case, an opportune diagnosis of RCC allowed for curative treatment and subsequent blood pressure control. Furthermore, this report underscores the pivotal role of internal medicine in the comprehensive evaluation of complex patients, contributing to improved morbidity and mortality.
